# Circulating Phylloquinone and the Risk of Four Female-Specific Cancers: A Mendelian Randomization Study

**DOI:** 10.3390/nu16213680

**Published:** 2024-10-29

**Authors:** Melaku Yalew, Anwar Mulugeta, Amanda L. Lumsden, Iqbal Madakkatel, S. Hong Lee, Martin K. Oehler, Johanna Mäenpää, Elina Hyppönen

**Affiliations:** 1 Australian Centre for Precision Health, Unit of Clinical and Health Sciences, University of South Australia, Adelaide, SA 5001, Australia; 2 South Australia Health and Medical Research Institute, Adelaide, SA 5000, Australia; 3 Department of Public Health, College of Medicine and Health Sciences, Injibara University, Injibara P.O. Box 6040, Ethiopia; 4 Department of Pharmacology and Clinical Pharmacy, College of Health Sciences, Addis Ababa University, Addis Ababa P.O. Box 9086, Ethiopia; 5 UniSA Allied Health & Human Performance, University of South Australia, Adelaide, SA 5001, Australia; 6 Department of Gynecological Oncology, Royal Adelaide Hospital, Adelaide, SA 5000, Australia; martin.oehler@adelaide.edu.au; 7 Adelaide Medical School, Robinson Research Institute, University of Adelaide, Adelaide, SA 5006, Australia; 8 Faculty of Medicine and Medical Technology, Tampere University, 33014 Tampere, Finland; 9 Cancer Centre, Tampere University and University Hospital, 33520 Tampere, Finland

**Keywords:** Mendelian randomization, vitamin K_1_, circulating phylloquinone, female-specific cancers

## Abstract

Background: Observational studies have linked vitamin K and cancer, but the causality of this association remains unknown. This Mendelian randomization (MR) study aims to investigate the association between circulating phylloquinone (vitamin K_1_) levels and four female-specific cancers. Methods: We used four single-nucleotide polymorphisms (SNPs) to instrument phylloquinone, with the reported F-statistic 16.00–28.44 for all variants. SNP–outcome associations were obtained from consortia meta-analyses, UK Biobank, and the FinnGen database (up to 145,257/419,675, 27,446/362,324, 15,181/591,477, and 2211/320,454 cases/controls for breast, ovarian, endometrial, and cervical cancer, respectively). Analyses were conducted using five complementary MR methods including pleiotropy robust approaches. The MR Egger intercept test, MR PRESSO global test and leave-one-out analyses were used to test for and identify pleiotropic variants. Results: The relevance of the instrument was validated by positive control analyses on coagulation factor IX (*p* = 0.01). However, the main MR analysis and all sensitivity analyses were consistently supportive of a null association between phylloquinone and all four cancers (*p* > 0.05 for all analyses, across all methods). MR-PRESSO did not detect outlying variants, and there was no evidence of horizontal pleiotropy relating to any cancer outcome (*p*_intercept_ > 0.26 for all). Conclusions: We found no evidence for an association between genetically predicted circulating phylloquinone levels and the risk of four female-specific cancers.

## 1. Introduction

Vitamin K represents a group of fat-soluble vitamins that come in two natural forms, phylloquinone (K_1_) and menaquinone (K_2_) [1]. Phylloquinone is the most common form in the circulation, and its levels reflect dietary intakes, primarily from green leafy vegetables [2,3,4,5], while menaquinone is typically produced by intestinal bacteria or obtained from fermented foods [6,7,8]. Circulating phylloquinone levels are also affected by common genetic variations, and a recent meta-analysis identified five genetic loci variants associated with circulating concentrations [9]. Once phylloquinone enters the metabolism, it can be converted to menaquinone [2,10,11], and both forms are believed to play a crucial physiological role in regulating blood coagulation [12,13,14]. However, there is also some evidence supporting the role of phylloquinone in many chronic diseases, including diabetes, cardiovascular disease, and cancer [15,16,17,18,19], and even for mortality risk [20,21,22], highlighting the need for further research in this field.

Promising evidence from secondary analyses in a randomized placebo-controlled trial suggests that phylloquinone supplementation can lower the risk of cancer [3]. Observational studies have also linked higher phylloquinone intakes to lower cancer mortality [21,22], and there is evidence to suggest a lowering of cancer risk in some [17] but not all studies [23,24], potentially depending on the type of cancer. For menaquinone intake and studies using vitamin K antagonists, evidence is mixed, with studies reporting increases as well as decreases in cancer risk [23,24,25,26,27]. However, experimental studies on several cancer cell lines have shown that vitamin K derivatives can exert inhibitory effects on cell growth, with evidence to suggest that phylloquinone also exerts an antiproliferative capacity [28,29,30,31,32]. While this may also have relevance for the risk of female-specific cancers, so far, there is little research looking into the possible role of phylloquinone.

In this study, we used a Mendelian randomization (MR) approach to investigate genetic evidence for an association between circulating phylloquinone levels and four female-specific cancers, including breast, ovarian, endometrial, and cervical cancers. MR is a form of instrumental variable analysis that can help to establish proof of principle evidence for a causal association, as it is less vulnerable to bias from confounding and reverse causation, which commonly affect findings from observational studies [33]. We combine data from several consortia meta-analyses, UK Biobank (UKB), and FinnGen resources, with this large-scale study including over 145,000 cases and 419,000 controls for the analysis of breast cancer.

## 2. Materials and Methods

### 2.1. Data Source and Selection of Genetic Instruments

A meta-analysis of available genome-wide association studies (GWASs) by the Cohorts for Heart and Aging Research in Genomic Epidemiology (CHARGE) Consortium Nutrition Working Group involving 2138 individuals of European ancestry identified 11 significant single-nucleotide polymorphisms (SNPs) from five loci associated with circulating phylloquinone at *p* < 1 × 10^−6^ [9]. Given that SNPs from each locus are in linkage disequilibrium (r^2^ > 0.9), we selected one SNP from each of the five loci. Additionally, we excluded one SNP (rs964184) that was strongly associated with triglycerides in the primary GWAS, as performed in prior studies [34,35,36]. Of the four SNPs used, a link with phylloquinone metabolism has been established for two variants: rs2108622 is a *CYP4F2* variant which functions as phylloquinone oxidase [37,38], while rs2192574 is a variant on the *CTNNA2* locus associated with bone mineral concentration via activation of osteocalcin (vitamin K-dependent protein) [39]. In the GWAS, circulating phylloquinone (nmol/L) was natural log-transformed, and the analysis was adjusted for age, sex, principal components, and other study-specific covariates, with the association between the variants and phylloquinone further confirmed in analyses that adjusted for triglyceride levels [9]. We validated the relevance of the instruments using positive control analyses on coagulation factor IX measurement [40], given the well-known role of vitamin K in blood coagulation [12].

### 2.2. Cancer Outcomes

We used information on breast cancer (BC), ovarian cancer (OC), endometrial cancer (EC), and cervical cancer (CC) as primary outcomes, with summary-level genetic data obtained from respective genetic consortia where available. We obtained data for BC from the Breast Cancer Association Consortia (BCAC, 122,977 cases and 105,974 controls) [41], for OC from the Ovarian Cancer Association Consortia (OCAC, 25,509 cases and 40,941 controls) [42], and for EC from the Endometrial Cancer Association Consortia (ECAC, 12,906 cases and 108,979 controls) [43]. For cervical cancer genetic associations, data were not available from consortia meta-analyses. We further included data on all four cancers from the UKB and FinnGen for the meta-analysis. Where possible, analyses were also conducted to consider cancer subtypes, including estrogen receptor-positive (ER+) and estrogen receptor-negative (ER−) BC-specific data from BCAC and FinnGen, OC subtype data mainly from OCAC, and endometrioid and non-endometrioid EC subtypes from ECAC. An overview of the sources of summary-based data used, which are accessible in the OpenGWAS platform [44], is presented in Table S1.

### 2.3. Statistical Analysis

The two-sample MR approach was used to investigate the association between genetically determined circulating phylloquinone levels and female-specific cancers. The strength of the genetic instruments was assessed using F-statistics, with a threshold of ≥10 considered sufficient to avoid weak instrument bias [45]. We extracted the SNP-specific cancer association estimates from the respective outcome data sources. When the genetic instrument was not found in the outcome data, the estimates for proxy variants (r^2^ > 0.8) were taken. We set the significance level (Type I error rate) at 0.05 and estimated the statistical power for this study using the method proposed by Burgess [46], assuming beta varied from 0.1 to 0.7 for weak and strong associations, respectively. Inverse variance-weighted (IVW) MR was used as the primary method of analysis [47], with estimates meta-analyzed across independent data sources. A random effects model was used in the meta-analysis to account for population variations across the studies. A series of statistical tests/sensitivity analyses, including weighted median [48], weighted mode [49], MR Egger [50], MR-PRESSO (pleiotropy residual sum and outlier) [51], and leave-one-out approaches were performed to investigate the sensitivity to potential violations in MR assumptions. All statistical tests were two-tailed, and analyses were performed using R version 4.3.2.

## 3. Results

### 3.1. Instrument Strength and Validation

The characteristics of four independent SNPs used to instrument circulating phylloquinone are presented in Table 1. The F-statistics for these SNPs ranged from 16.00 to 28.44, which shows that bias from weak instruments is unlikely. Each SNP explained approximately 0.7 to 1.3% of the variation in circulating phylloquinone. We performed a positive control analysis to validate the genetic instruments, using coagulation factor IX measurements as the outcome. Of the four SNPs, none were individually associated with coagulation factor IX levels; however, all estimates were directionally consistent, and IVM MR analysis confirmed the expected positive association between circulating phylloquinone levels and coagulation factor IX measurements (beta 0.10, 95% CI 0.02, 0.17) (Table 1 and Table S2).

### 3.2. Mendelian Randomization Analysis

**Figure 1:**
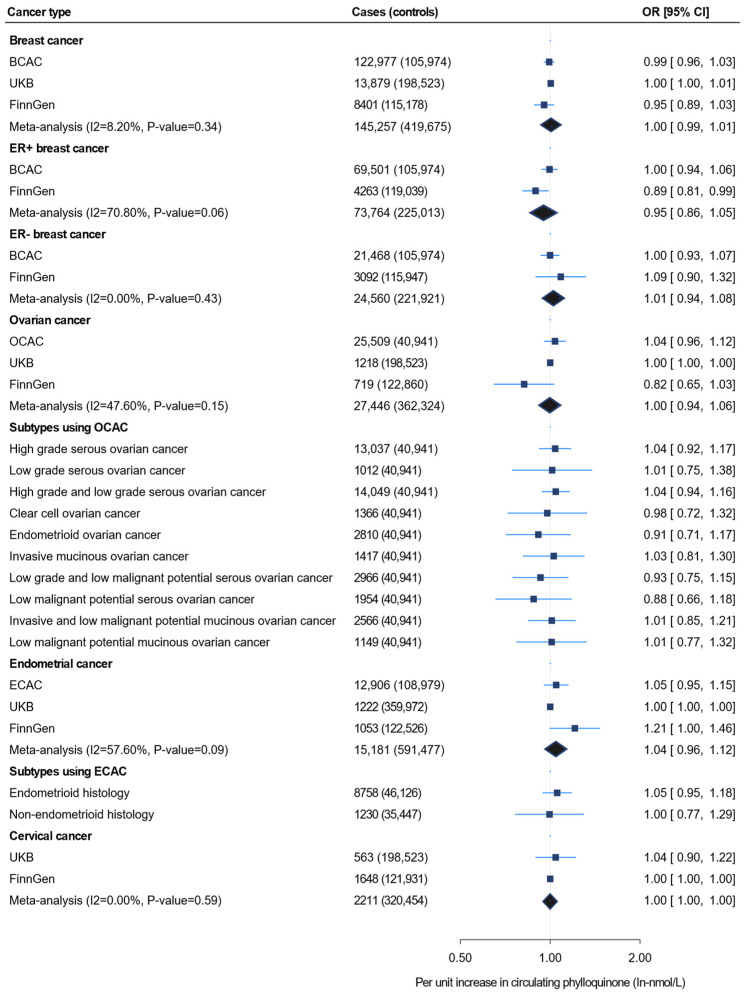
Inverse-variance weighted Mendelian randomization for the association between unit increase in circulating phylloquinone level (In-nmol/L) and four female-specific cancers, including the subtypes. UKB = UK Biobank; OCAC = Ovarian Cancer Association Consortium; BCAC = Breast Cancer Association Consortium; ECAC = Endometrial Cancer Association Consortium.

Figure 1 shows the IVW MR findings for the association between circulating phylloquinone and four female-specific cancers, including the subtypes. The effect estimates for the same risk allele in each SNP across all cancer types are provided in Table S4. Our analysis using 122,979 BC cases from BCAC did not show an association between genetically determined circulating phylloquinone and BC [odds ratio (OR) 0.99, 95% confidence interval (CI) 0.96, 1.03]. Similar findings were observed in analyses using 13,879 cases from the UKB and 8401 cases from FinnGen, as well as in the combined meta-analysis of 145,257 cases from the three data sources (Figure 1, Table S5).

There was also no evidence for an association between circulating phylloquinone and either ER + or ER − BC using the data from consortia meta-analyses. A borderline negative association was observed between circulating phylloquinone and ER + BC in the FinnGen population (OR 0.89, 95% CI 0.81, 0.99); however, this association was not robustly confirmed by the meta-analysis (OR 0.95, 95% CI 0.86, 1.05) (Figure 1). Analysis of 25,509 OC cases from OCAC did not support an association between genetically determined circulating phylloquinone and OC (OR 1.04, 95% CI 0.96, 1.12). Similar null findings were observed in the analyses using UKB (OR 1.00, 95% CI 0.1.00, 1.00) and FinnGen (OR 0.82, 95% CI 0.65, 1.03). These findings remained consistent after aggregating the OC cases from all three of these independent outcome data sources (cases = 27,446) in the meta-analysis (Figure 1). Similarly, no evidence of association was shown in the OC subtype analysis. Genetically determined circulating phylloquinone also showed no association with EC in analyses using ECAC, UKB, and FinnGen (OR 1.05, 95% CI 0.95, 1.15; OR 1.00, 95% CI 1.00, 1.00; and OR 1.21, 95% CI 1.00, 1.46, respectively), nor with its endometrioid and non-endometrioid histological subtypes. Furthermore, genetically determined circulating phylloquinone levels were not associated with cervical cancer in the UKB, FinnGen, or the meta-analysis (Figure 1, Table S5).

For all cancer outcomes, the results from weighted median MR, weighted mode MR, MR-Egger, and MR-PRESSO were consistent with the findings of IVW MR. The MR-Egger intercept test did not identify evidence for horizontal pleiotropy relating to any cancer outcome (*p*_intercept_ > 0.26 for all), and analyses using the MR-PRESSO did not detect outlying variants. Full detailed results of sensitivity analyses, including leave-one-out analyses for all cancer subtypes, are presented in the Supplementary Materials (Table S6 and Figure S1). Furthermore, the MR analysis was repeated using genetic instrument–phylloquinone association estimates adjusted for triglyceride levels (Table S7) and only using the two clinically relevant SNPs (rs2192574 and rs2108622) (Table S8), with the null associations remaining unchanged.

### 3.3. Power Analysis

We assessed the statistical power for our ability to detect an association with each cancer type. Power was estimated based on an alpha level of 0.05, assuming that the total variance (R^2^) explained by the instruments for phylloquinone is 4.7% [36]. The minimum detectable OR by our meta-analyses varied from 1.04 (breast cancer) to 1.35 (cervical cancer), with full details given in Table S9.

## 4. Discussion

Observational studies on the role of vitamin K in cancer have provided mixed results, while for phylloquinone (K_1_), there is some randomized controlled trial evidence supporting a potential benefit [3]. In this large-scale MR study, we tested for associations between genetically determined circulating phylloquinone levels and female-specific cancers, including on breast, ovarian, endometrial, and cervical cancers. We found no evidence for a causal link between phylloquinone levels and any of the four cancers, with this finding being robust across all sensitivity analyses and with no evidence of horizontal pleiotropy.

Our null finding for breast cancer is in line with a previous prospective cohort study of 78,209 women (2286 cases) which also reported null associations for total dietary vitamin K intake and phylloquinone intake with incident breast cancer [24]. However, this previous study observed some evidence for an increased breast cancer risk by higher menaquinone intake, with an adverse association also observed with breast cancer mortality [24]. There is mechanistic evidence to suggest that phylloquinone and menaquinone can exert differential effects in breast cancer cells [52]. Indeed, if anything, for phylloquinone concentrations, our analyses provided tentative evidence for a potential benefit of higher serum concentrations on ER+ breast cancer.

For the other female-specific cancers included in our study, data from earlier studies in humans are sparse. Some previous studies investigating vitamin K or phylloquinone associations with cancer risk have focused on overall cancer risk or cancer mortality, with at least one observational study [22] and one clinical trial [3] supporting potential benefits.

A large number of observational studies have investigated the role of vitamin K in specific types of cancers, with the results suggesting both increased and decreased risks linked to higher dietary intakes [17,23,24]. There are also several studies investigating the use of vitamin K antagonists, with a systematic review and meta-analysis concluding a reduction in cancer risk [25]. For phylloquinone intake, there is some evidence for a protective role against pancreatic cancer, overall cancer risk, and mortality [3,17]. While studies looking into phylloquinone have not provided evidence suggesting a need for caution, mixed findings from studies looking at other forms of vitamin K (mainly menaquinone) or vitamin K antagonists may reflect the complexity of mechanisms by which vitamin K may exert effects on cancer risk.

A review of studies investigating the role of vitamin K in selected female cancers outlines several mechanisms through which vitamin K may exert its effects in breast, cervical, and ovarian cancer, including those related to antiproliferation, induction of mitochondrial dysfunction, generation of reactive oxygen species, activation of apoptotic pathways/apoptosis, photosensitizing effects, synergistic effects with vitamin C, and cell adhesion inhibition [31]. Continued research is required to fully understand the complexity of the role of vitamin K in cancer prevention and treatment.

Our study has several notable strengths. To the best of our knowledge, this is the first MR study to assess the association between genetically determined phylloquinone levels and female-specific cancers. Additionally, we enhanced the statistical power by meta-analyzing the independent MR results from larger cohort studies such as the UKB and FinnGen, integrating these with data from the relevant consortia. While it is very difficult to ‘prove’ an absence of an association, our analyses appeared relatively well powered for the major cancer types. According to the power calculation, we had sufficient power to detect a 4% difference in breast cancer (8% for ovarian cancer), which is similar to the observed effect on type 2 diabetes and using the same instruments (OR 0.93) [36]. We used a threshold of *p* < 1 × 10^−6^ for the genetic instruments, which is more lenient than the commonly accepted threshold for genome-wide analyses (*p* < 5 × 10^−8^); however, all the instruments have F-statistics greater than 10 and we validated these instruments using a positive control analysis. Finally, the two main potential biases that can impact the results of MR studies are population stratification and pleiotropy. In our study, these biases are unlikely to have a major influence on our findings. By including only individuals of European ancestry, we effectively minimized the risk of population stratification, ensuring that our results are not confounded by ancestral differences. Furthermore, we conducted several sensitivity analyses to confirm the robustness of our findings. These analyses were consistent with our main results and showed no evidence of horizontal pleiotropy, providing additional reassurance that our conclusions are reliable.

However, this study is not without limitations. Firstly, our findings are applicable only to individuals of European ancestry, as other ancestries are not represented in our sample. This limits the generalizability of our results to more diverse populations. Secondly, the sample size from which the SNPs were drawn was relatively small, and only four SNPs were used to instrument phylloquinone. Consequently, the null associations in the subtypes might be because of low statistical power since our analyses were only powered for overall cancers.

## 5. Conclusions

Although vitamin K has been suggested to exert an effect on cancer through multiple mechanisms, this MR study found no evidence of an association between genetically predicted phylloquinone levels and the risk of female-specific cancers. Despite these null findings, future research should aim to identify additional SNPs associated with phylloquinone and validate our findings with larger sample sizes to explore the relationships.

## Figures and Tables

**Table 1:** The selected four SNPs used to instrument phylloquinone with their effect estimates *.

SNPs	Chr	Nearest Gene	Effect Allele	Alternative Allele	Phylloquinone					Coagulation Factor IX		
					Beta ^a^	SE	*p*-Value	R^2^	F-Statistics	Beta ^b^	SE	*p*-Value
rs2192574	2	*CTNAA2*	C	T	0.28	0.06	1.82 × 10^−6^ **	0.010	21.78	0.020	0.020	0.34
rs4122275	5	*CDO1*	G	A	0.68	0.17	4.76 × 10^−5^ ***	0.007	16.00	0.078	0.051	0.13
rs4645543	8	*KCNK9*	C	T	0.42	0.08	2.0 × 10^−7^	0.013	27.56	0.046	0.031	0.14
rs2108622	19	*CYP4F2*	T	C	0.16	0.03	8.78 × 10^−7^	0.013	28.44	0.015	0.015	0.32

Note. * = Estimates taken from CHARGE Nutrition working group meta-analyses [9]. SE = standard error. ^a^ = the coefficient indicates higher phylloquinone (ln-nmol/L) per additional copy of the effect allele, adjusted for age, sex, and study-specific covariates. ^b^ = the coefficient indicates a change in coagulation factor IX (logarithmically transformed normalized protein expression (NPX)) per additional copy of the effect allele. ** = the *p*-value passes the threshold (<1 × 10^−6^) for selecting the SNPs after adjusting additionally for triglyceride and vegetable intake. *** = the *p*-values pass the threshold (<1 × 10^−6^) for selecting the SNPs after adjusting additionally for triglyceride. The extended genetic variant associations with circulating phylloquinone under different adjustment models (as applied in the discovery GWAS) are found in Table S3).

## Data Availability

All the data analyzed/used in the current study are available in MRC IEU OpenGWAS, and the code for the analysis can be shared upon request to the authors.

## Supplementary Materials

The following supporting information can be downloaded at https://www.mdpi.com/article/10.3390/nu16213680/s1: Table S1: Characteristics of the outcome data sources, and number of instruments used for each variant–outcome association; Table S2: Positive control analyses of phylloquinone on coagulation factor IX with SNP association estimates for the instrument taken from models with and without adjustment for triglycerides; Table S3: Genetic variant associations with circulating phylloquinone, including estimates from the discovery GWAS, along with the corresponding R-squared and F-statistics; Table S4: Individual SNP association estimates across all data sources and cancer types; Table S5: Mendelian randomization analyses across outcome data sources and cancer types in fixed and random effect meta-analyses; Table S6: Mendelian randomization analyses of phylloquinone on female-specific cancers using different methods; Table S7: Mendelian randomization analyses of phylloquinone on female-specific cancers using different methods with SNP–phylloquinone association estimates taken from models adjusting for triglycerides; Table S8: Mendelian randomization analyses of phylloquinone on female-specific cancers using different methods and restricting the instrument to two clinically relevant SNPs (rs2192574 near to *CTNAA2* gene and rs2108622 near to *CYP4F2*); Table S9: Statistical power and minimum detectable odds ratio at 80% power for the association between phylloquinone and four female-specific cancers; Figure S1: Leave-one-out sensitivity analysis for the association between circulating phylloquinone, four female-specific cancers, and the coagulation factor IX measurement as the positive control.
