# Supplementary material for: Circulating Phylloquinone and the Risk of Four Female-Specific Cancers: A Mendelian Randomization Study
Source: Nutrients. 2024 Oct 29;16(21):3680. doi: 10.3390/nu16213680 (PMC11547380; doi:10.3390/nu16213680)
Supplement: Supplementary file 1 [file nutrients-16-03680-s001.zip › nutrients-3245373-supplementary.pdf]

Supplementary materials

Contents

Table S1: Characteristics of the outcome data sources, and number of instruments used 2

Table S2: Positive control analyses of phylloquinone on coagulation factor IX with SNP-association estimate for the instrument taken from models with and without adjustment for triglycerides............. 3

Table S3: Genetic variant associations with circulating phylloquinone, including estimates from the discovery GWAS, along with the corresponding R-squared and F-statistics.. 4

Table S4: Individual SNP-association estimates across all data sources and cancer types. 5

Table S5: Mendelian randomization analyses across outcome data sources and cancer types in random effect meta-analyses.. 7

Table S6: Mendelian randomization analyses of phylloquinone on female-specific cancers using different methods. 8

Table S7: Mendelian randomization analyses of phylloquinone on female-specific cancers using different methods with SNP–phylloquinone association estimates taken from models adjusting for triglycerides.. 9

Table S8: Mendelian randomization analyses of phylloquinone on female-specific cancers using different methods and restricting the instrument to two clinically relevant SNPs (rs2192574 near to *CTNAA2* gene and rs2108622 near to *CYP4F2*). 10

Table S9: Statistical power and minimum detectable odds ratio at 80% power for the association between phylloquinone and four female-specific cancers. 11

Figure S1: Leave-one-out sensitivity analysis for the association between circulating phylloquinone, four female-specific cancers, and the coagulation factor IX measurement as the positive control. 12

Table S1: Characteristics of the outcome data sources, and number of instruments used

Outcome	Data source	Cases	Sample size	SNPs	Authors/Year
Breast cancer	BCAC	122,977	228,951	4	Michailidou K et al./2017
	UKB	13,879	212,402	3	Burrows et al./2021
	FinnGen	8,401	123,579	4	NA
Ovarian cancer	OCAC	25,509	66,450	4	Phelan et al./2017
	UKB	1,218	199,741	3	Burrows et al./2021
	FinnGen	719	123,579	4	NA
Endometrial cancer	ECAC	12,906	121,885	4	O'Mara TA et al./2018
	UKB	1,222	361,194	4	Neale lab et al./2018
	FinnGen	1,053	123,579	4	NA
Cervical Cancer	UKB	563	199,086	2	Burrows et al./2021
	FinnGen	1,648	123,579	4	NA
Positive control analyses					
Coagulation factor IX*	Fenland cohort	-	10,708	4	Pietzner M et al.

*= positive control outcomes, NA=FinnGen cohort release 3 (finn-b-C3) available in MR-base, UKB=UK-Biobank, OCAC= Ovarian Cancer Association Consortium, BCAC= Breast Cancer Association Consortium, ECAC= Endometrial Cancer Association Consortium.

Table S2: Positive control analyses of phylloquinone on coagulation factor IX with SNP-association estimate for the instrument taken from models with and without adjustment for triglycerides.

				MR IVW		Weighted median		Weighted mode		MR-Egger		
**Outcome**	**SNPs**	**Estimates**	**Sample size**	Beta (95% CI)	P-value	Beta (95% CI)	P-value	Beta (95% CI)	P-value	Beta (95% CI)	P-value	P-intercept
Coagulation factor IX measurement	4	Taken before triglyceride adjustment	10,708	0.10 (0.02, 0.17)	0.013	0.10 (0.01, 0.19)	0.027	0.11 (-0.63, 0.85)	0.156	0.12 (-0.24, 0.48)	0.301	0.819
		Taken after triglyceride adjustment	10,708	0.10 (0.02, 0.17)	0.013	0.09 (0.00, 0.18)	0.05	0.09 (-0.07, 0.26)	0.05	0.10 (-0.22, 0.43)	0.08	0.922

Table S3: Genetic variant associations with circulating phylloquinone, including estimates from the discovery GWAS, along with the corresponding R-squared and F-statistics

					Model 1					Model 2					Model 3				
SNP	Chr	Nearest gene	Effect allele	Alternative allele	Beta	SE	P-value	R^2	F-stat	Beta	SE	P-value	R^2	F-stat	Beta	SE[c]	P-value	R^2	F-stat
rs2192574	2	CTNAA2	C	T	0.28	0.06	1.82E-06	0.010	21.78	0.28	0.06	1.49E-06	0.010	21.78	0.29	0.06	8.2E-07	0.011	23.36
rs4122275	5	CDO1	G	A	0.68	0.17	4.76E-05	0.007	16.00	0.81	0.16	4.31E-07	0.012	25.63	0.78	0.17	3.9E-06	0.010	21.05
rs4645543	8	KCNK9	C	T	0.42	0.08	2.00E-07	0.013	27.56	0.35	0.08	5.69E-06	0.010	19.14	0.35	0.08	1.6E-05	0.010	19.14
rs2108622	19	CYP4F2	T	C	0.16	0.03	8.78E-07	0.013	28.44	0.16	0.03	2.90E-07	0.013	28.44	0.16	0.03	9.7E-07	0.013	28.44

Chr=Chromosome, SE=Standard Error. Model 1 is adjusted for age, sex, and study-specific covariates, Model 2 is adjusted for age, sex, study-specific covariates, and triglycerides, and Model 3 is adjusted for age, sex, study-specific covariates, triglycerides, and vegetable intake.

Table S4: Individual SNP-association estimates across all data sources and cancer types.

SNP	Chr	logOR	SE	P-value	Sample size	Cancer type	Data source
rs2192574	2	0.00240	0.01010	0.80830	228951	Breast cancer	BCAC
rs4122275	5	-0.02490	0.02600	0.33830	228951	Breast cancer	BCAC
rs4645543	8	0.01010	0.01550	0.51580	228951	Breast cancer	BCAC
rs2108622	19	-0.00650	0.00700	0.35080	228951	Breast cancer	BCAC
rs2192574	2	0.00029	0.00115	0.80000	212402	Breast cancer	UKB
rs4122275	5	0.00321	0.00277	0.25000	212402	Breast cancer	UKB
rs4645543	8	0.00214	0.00176	0.22000	212402	Breast cancer	UKB
rs2192574	2	-0.02180	0.02300	0.34380	123,573	Breast cancer	FinnGen
rs4122275	5	-0.01990	0.04030	0.62180	123,573	Breast cancer	FinnGen
rs4645543	8	-0.00770	0.02910	0.79180	123,573	Breast cancer	FinnGen
rs2108622	19	-0.02640	0.02170	0.22380	123,573	Breast cancer	FinnGen
rs2192574	2	0.00590	0.01210	0.62630	175475	ER+ Breast cancer	BCAC
rs4122275	5	-0.02960	0.03090	0.33830	175475	ER+ Breast cancer	BCAC
rs4645543	8	0.02380	0.01850	0.19820	175475	ER+ Breast cancer	BCAC
rs2108622	19	-0.01290	0.00840	0.12130	175475	ER+ Breast cancer	BCAC
rs2192574	2	-0.02660	0.03060	0.38390	123,302	ER+ Breast cancer	FinnGen
rs4122275	5	-0.11990	0.05330	0.02431	123,302	ER+ Breast cancer	FinnGen
rs4645543	8	-0.01620	0.03870	0.67650	123,302	ER+ Breast cancer	FinnGen
rs2108622	19	-0.01890	0.02890	0.51460	123,302	ER+ Breast cancer	FinnGen
rs2192574	2	0.00100	0.01840	0.95490	127442	ER- Breast cancer	BCAC
rs4122275	5	-0.02790	0.04790	0.55930	127442	ER- Breast cancer	BCAC
rs4645543	8	0.00380	0.02850	0.89460	127442	ER- Breast cancer	BCAC
rs2108622	19	0.00440	0.01270	0.72980	127442	ER- Breast cancer	BCAC
rs2192574	2	-0.01620	0.03550	0.64700	119,039	ER- Breast cancer	FinnGen
rs4122275	5	0.19470	0.06270	0.00189	119,039	ER- Breast cancer	FinnGen
rs4645543	8	-0.02040	0.04500	0.64980	119,039	ER- Breast cancer	FinnGen
rs2108622	19	-0.01590	0.03350	0.63430	119,039	ER- Breast cancer	FinnGen
rs2192574	2	0.03155	0.02172	0.14630	66450	Ovarian cancer	OCAC
rs4122275	5	0.03899	0.05408	0.47090	66450	Ovarian cancer	OCAC
rs4645543	8	-0.00677	0.03247	0.8348	66450	Ovarian cancer	OCAC
rs2108622	19	-0.00289	0.01463	0.84340	66450	Ovarian cancer	OCAC
rs2192574	2	0.00003	0.00037	0.94000	199741	Ovarian cancer	UKB
rs4122275	5	0.00007	0.00090	0.94000	199741	Ovarian cancer	UKB
rs4645543	8	0.00022	0.00057	0.71000	199741	Ovarian cancer	UKB
rs2192574	2	-0.10450	0.07080	0.14020	123,579	Ovarian cancer	FinnGen
rs4122275	5	-0.11980	0.12360	0.33260	123,579	Ovarian cancer	FinnGen
rs4645543	8	-0.09660	0.08970	0.28120	123,579	Ovarian cancer	FinnGen
rs2108622	19	0.03990	0.06710	0.55180	123,579	Ovarian cancer	FinnGen
rs2192574	2	0.05681	0.02573	0.02725	53978	High grade serous ovarian cancer	OCAC
rs4122275	5	0.00318	0.06380	0.96020	53978	High grade serous ovarian cancer	OCAC
rs4645543	8	-0.00333	0.03883	0.93170	53978	High grade serous ovarian cancer	OCAC
rs2108622	19	-0.01342	0.01740	0.44060	53978	High grade serous ovarian cancer	OCAC
rs2192574	2	0.00223	0.07804	0.97720	41953	Low grade serous ovarian cancer	OCAC
rs4122275	5	0.08045	0.20390	0.69320	41953	Low grade serous ovarian cancer	OCAC
rs4645543	8	-0.14190	0.11220	0.20620	41953	Low grade serous ovarian cancer	OCAC
rs2108622	19	0.06815	0.05211	0.19090	41953	Low grade serous ovarian cancer	OCAC
rs2192574	2	0.05313	0.02501	0.03366	54990	High grade and low grade serous ovarian cancer	OCAC
rs4122275	5	0.01395	0.06221	0.82260	54990	High grade and low grade serous ovarian cancer	OCAC
rs4645543	8	-0.0109	0.03764	0.77210	54990	High grade and low grade serous ovarian cancer	OCAC
rs2108622	19	-0.00565	0.01689	0.73780	54990	High grade and low grade serous ovarian cancer	OCAC
rs2192574	2	0.09104	0.06451	0.15820	42307	Clear cell ovarian cancer	OCAC
rs4122275	5	-0.11550	0.15700	0.46180	42307	Clear cell ovarian cancer	OCAC
rs4645543	8	0.02335	0.09804	0.81170	42307	Clear cell ovarian cancer	OCAC
rs2108622	19	-0.06855	0.04458	0.12410	42307	Clear cell ovarian cancer	OCAC
rs2192574	2	-0.06293	0.04785	0.18850	43751	Endometrioid ovarian cancer	OCAC
rs4122275	5	0.02736	0.11730	0.81560	43751	Endometrioid ovarian cancer	OCAC
rs4645543	8	-0.13310	0.06774	0.04946	43751	Endometrioid ovarian cancer	OCAC
rs2108622	19	0.04070	0.03157	0.19740	43751	Endometrioid ovarian cancer	OCAC
rs2192574	2	-0.00961	0.06425	0.88110	42358	Invasive mucinous ovarian cancer	OCAC
rs4122275	5	0.06879	0.16270	0.67250	42358	Invasive mucinous ovarian cancer	OCAC
rs4645543	8	-0.07131	0.09312	0.44380	42358	Invasive mucinous ovarian cancer	OCAC
rs2108622	19	0.05110	0.04358	0.24100	42358	Invasive mucinous ovarian cancer	OCAC
rs2192574	2	0.02850	0.04715	0.54540	43907	Low grade and low malignant potential serous ovarian cancer	OCAC
rs4122275	5	0.07103	0.11820	0.54790	43907	Low grade and low malignant potential serous ovarian cancer	OCAC
rs4645543	8	-0.12620	0.06765	0.06221	43907	Low grade and low malignant potential serous ovarian cancer	OCAC
rs2108622	19	-0.03399	0.03191	0.28680	43907	Low grade and low malignant potential serous ovarian cancer	OCAC

rs2192574	2	0.03561	0.05722	0.53370	42895	Low malignant potential serous ovarian cancer	OCAC
rs4122275	5	0.05637	0.14060	0.68860	42895	Low malignant potential serous ovarian cancer	OCAC
rs4645543	8	-0.12470	0.08188	0.12770	42895	Low malignant potential serous ovarian cancer	OCAC
rs2108622	19	-0.08337	0.039080	0.03290	42895	Low malignant potential serous ovarian cancer	OCAC
rs2192574	2	0.02101	0.04891	0.66750	43507	Invasive and low malignant potential mucinous ovarian cancer	OCAC
rs4122275	5	0.05436	0.12270	0.65770	43507	Invasive and low malignant potential mucinous ovarian cancer	OCAC
rs4645543	8	-0.04731	0.07251	0.51410	43507	Invasive and low malignant potential mucinous ovarian cancer	OCAC
rs2108622	19	0.00327	0.03332	0.92170	43507	Invasive and low malignant potential mucinous ovarian cancer	OCAC
rs2192574	2	0.04923	0.07248	0.49690	42090	Low malignant potential mucinous ovarian cancer	OCAC
rs4122275	5	0.03149	0.17850	0.86000	42090	Low malignant potential mucinous ovarian cancer	OCAC
rs4645543	8	0.03217	0.11190	0.77380	42090	Low malignant potential mucinous ovarian cancer	OCAC
rs2108622	19	-0.05772	0.04937	0.24230	42090	Low malignant potential mucinous ovarian cancer	OCAC
rs2192574	2	0.01713	0.02400	0.47532	121885	Endometrial cancer	ECAC
rs4122275	5	0.05368	0.06739	0.42573	121885	Endometrial cancer	ECAC
rs4645543	8	0.01346	0.03699	0.71585	121885	Endometrial cancer	ECAC
rs2108622	19	0.00079	0.01692	0.96289	121885	Endometrial cancer	ECAC
rs2192574	2	0.00009	0.00021	0.65423	361194	Endometrial cancer	UKB
rs4122275	5	0.00044	0.00051	0.38302	361194	Endometrial cancer	UKB
rs4645543	8	0.00034	0.00032	0.28497	361194	Endometrial cancer	UKB
rs2108622	19	0.00006	0.00015	0.70584	361194	Endometrial cancer	UKB
rs2192574	2	0.02950	0.05910	0.61790	123,579	Endometrial cancer	FinnGen
rs4122275	5	0.13530	0.10310	0.18950	123,579	Endometrial cancer	FinnGen
rs4645543	8	0.13390	0.07490	0.07385	123,579	Endometrial cancer	FinnGen
rs2108622	19	-0.01780	0.05590	0.75000	123,579	Endometrial cancer	FinnGen
rs2192574	2	0.00993	0.02838	0.72635	54884	Endometrial cancer (endometrioid histology)	ECAC
rs4122275	5	0.02285	0.08000	0.77513	54884	Endometrial cancer (endometrioid histology)	ECAC
rs4645543	8	0.04160	0.04389	0.34318	54884	Endometrial cancer (endometrioid histology)	ECAC
rs2108622	19	0.00640	0.01997	0.74838	54884	Endometrial cancer (endometrioid histology)	ECAC
rs2192574	2	0.05185	0.06936	0.45471	36677	Endometrial cancer (non-endometrioid histology)	ECAC
rs4122275	5	-0.12394	0.17853	0.48752	36677	Endometrial cancer (non-endometrioid histology)	ECAC
rs4645543	8	-0.05907	0.10963	0.59000	36677	Endometrial cancer (non-endometrioid histology)	ECAC
rs2108622	19	0.02042	0.04858	0.67420	36677	Endometrial cancer (non-endometrioid histology)	ECAC
rs2192574	2	-0.02230	0.04740	0.63830	123,579	cervical cancer	FinnGen
rs4122275	5	-0.02030	0.08310	0.80660	123,579	cervical cancer	FinnGen
rs4645543	8	0.09360	0.05990	0.11790	123,579	cervical cancer	FinnGen
rs2108622	19	0.00850	0.04450	0.84820	123,579	cervical cancer	FinnGen
rs2192574	2	0.00003	0.00026	0.92000	199086	cervical cancer	UKB
rs4645543	8	-0.00006	0.00039	0.88000	199086	cervical cancer	UKB

SNP=Single Nucleotide Polymorphism, Chr=Chromosome, SE=Standard Error, ER+= Estrogen receptor positive breast cancer, ER-= Estrogen receptor negative breast cancer, UKB=UK-Biobank, OCAC= Ovarian Cancer Association Consortium, BCAC= Breast Cancer Association Consortium, ECAC= Endometrial Cancer Association Consortium.

Table S5: Mendelian randomization analyses across outcome data sources and cancer types in random effect meta-analyses.

		IVW			Weighted median			Weighted mode			MR-Egger			MR-PRESSO		
Cancer type	Data sources	OR	LCI	UCI	OR	LCI	UCI	OR	LCI	UCI	OR	LCI	UCI	OR	LCI	UCI
	BCAC	0.992	0.955	1.030	0.995	0.949	1.043	1.013	0.917	1.119	1.006	0.840	1.205	0.990	0.930	1.060
	UKB	1.004	0.999	1.008	1.005	0.999	1.010	1.005	0.991	1.020	1.008	0.920	1.105	NA		
	FinnGen	0.954	0.886	1.028	0.971	0.888	1.062	0.972	0.826	1.144	1.026	0.720	1.461	0.950	0.860	1.060
	meta-analysis	(I^2=8.20, p-value=0.34)			(I^2=0.00, p-value=0.69)			(I^2=0.00, p-value=0.91)			(I^2=0.00, p-value=0.84)			(I^2=0.00, p-value=0.51)		
Breast cancer	Random	1.002	0.99	1.010	1.005	0.999	1.010	1.005	0.991	1.019	1.009	0.931	1.092	0.979	0.926	1.034
	BCAC	0.995	0.938	1.055	1.001	0.948	1.057	1.025	0.896	1.172	1.040	0.763	1.417	0.990	0.870	1.130
	FinnGen	0.893	0.809	0.985	0.902	0.797	1.022	0.933	0.719	1.211	0.859	0.537	1.373	0.890	0.770	1.030
	meta-analysis	(I^2=70.80, p-value=0.06)			(I^2=55.80, p-value=0.13)			(I^2=0.00, p-value=0.53)			(I^2=0.00, p-value=0.51)			(I^2=12.20, p-value=0.29)		
ER+ breast cancer	Random	0.950	0.860	1.050	0.965	0.876	1.064	1.005	0.892	1.132	0.981	0.758	1.271	0.943	0.850	1.047
	BCAC	0.999	0.932	1.070	1.006	0.927	1.091	1.008	0.853	1.193	0.961	0.690	1.339	1.000	0.940	1.060
	FinnGen	1.085	0.895	1.315	0.952	0.806	1.125	0.948	0.67	1.341	1.379	0.643	2.956	1.080	0.710	1.650
	meta-analysis	(I^2=0.00, p-value=0.43)			(I^2=0.00, p-value=0.56)			(I^2=0.00, p-value=0.76)			(I^2=0.00, p-value=0.40)			(I^2=0.00, p-value=0.72)		
ER- breast cancer	Random	1.008	0.945	1.076	0.995	0.925	1.071	0.996	0.857	1.159	1.018	0.751	1.379	1.002	0.944	1.063
	OCAC	1.038	0.959	1.124	1.027	0.935	1.128	0.992	0.780	1.263	1.065	0.731	1.553	1.040	0.910	1.190
	UKB	1.000	0.999	1.002	1.000	0.998	1.002	1.000	0.996	1.005	1.000	0.971	1.030	NA		
	FinnGen	0.818	0.651	1.028	0.812	0.606	1.088	0.808	0.500	1.306	0.726	0.245	2.156	0.820	0.560	1.190
	meta-analysis	(I^2=47.60, p-value=0.15)			(I^2=11.30, p-value=0.32)			(I^2=0.00, p-value=0.68)			(I^2=0.00 p-value=0.80)			(I^2=26.20, p-value=0.24)		
Ovarian cancer	Random	0.999	0.940	1.060	1.000	0.970	1.031	1.000	0.995	1.004	1.000	0.971	1.030	0.988	0.816	1.197
	ECAC	1.047	0.955	1.148	1.049	0.944	1.166	1.057	0.846	1.319	1.089	0.694	1.708	1.050	0.980	1.120
	UKB	1.001	1.000	1.001	1.001	1.000	1.001	1.000	0.999	1.002	1.001	0.997	1.004	1.001	1.000	1.001
	FinnGen	1.210	1.000	1.464	1.216	0.954	1.550	1.231	0.797	1.903	1.429	0.577	3.541	1.2100	0.910	1.620
	meta-analysis	(I^2=57.60, p-value=0.09)			(I^2=38.00, p-value=0.20)			(I^2=0.00, p-value=0.57)			(I^2=0.00, p-value=0.57)			(I^2=45.60, p-value=0.16)		
Endometrial cancer	Random	1.040	0.960	1.120	1.025	0.962	1.092	1.000	0.999	1.002	1.001	0.998	1.005	1.020	0.972	1.071
	UKB	1.000	0.999	1.001												
	FinnGen	1.043	0.895	1.215												
	meta-analysis	(I^2=0.00, p-value=0.59)														
Cervical cancer	Random	1.000	0.999	1.001							NA					

OR=odds ratio, LCI= lower confidence interval, UCI=upper confidence interval, NA - not available due to an insufficient number of instruments to perform the specified MR method, ER+= Estrogen receptor positive breast cancer, ER- = Estrogen receptor negative breast cancer, UKB=UK-Biobank, OCAC= Ovarian Cancer Association Consortium, BCAC= Breast Cancer Association Consortium, ECAC= Endometrial Cancer Association Consortium.

Table S6: Mendelian randomization analyses of phylloquinone on female-specific cancers using different methods.

					MR IVW		Weighted median		Weighted mode		MR-Egger			MR-PRESSO		
Cancer types	SNPs	Cases	Total	Data sources	OR (95% CI)	P-val	OR (95% CI)	P-val	OR (95% CI)	P-val	OR (95% CI)	P-val	P-inter	OR (95% CI)	P-val	Global test P-val
Breast cancer	4	122,977	228,951	BCAC	0.992 (0.955, 1.030)	0.672	0.995 (0.949, 1.043)	0.829	1.013 (0.917, 1.119)	0.711	1.006 (0.840, 1.205)	0.900	0.742	0.990 (0.930, 1.060)	0.650	0.5911
Breast cancer	3	13,879	212,402	UKB	1.004 (0.999, 1.008)	0.129	1.005 (0.999, 1.010)	0.118	1.005 (0.991, 1.020)	0.280	1.008 (0.920, 1.105)	0.461	0.626	NA		
Breast cancer	4	8,401	123,579	FinnGen	0.954 (0.886, 1.028)	0.216	0.971 (0.888, 1.062)	0.525	0.972 (0.826, 1.144)	0.615	1.026 (0.720, 1.461)	0.787	0.426	0.950 (0.860, 1.060)	0.140	0.8241
ER+ Breast cancer	4	69,501	175,475	BCAC	0.995 (0.938, 1.055)	0.863	1.001 (0.948, 1.057)	0.971	1.025 (0.896, 1.172)	0.605	1.040 (0.763, 1.417)	0.640	0.559	0.990 (0.870, 1.130)	0.870	0.2455
ER+ Breast cancer	4	4,263	123,302	FinnGen	0.893 (0.809, 0.985)	0.024	0.902 (0.797, 1.022)	0.105	0.933 (0.719, 1.211)	0.460	0.859 (0.537, 1.373)	0.297	0.727	0.890 (0.770, 1.030)	0.040	0.6807
ER- Breast cancer	4	21,468	127,442	BCAC	0.999 (0.932, 1.070)	0.969	1.006 (0.927, 1.091)	0.891	1.008 (0.853, 1.193)	0.885	0.961 (0.690, 1.339)	0.660	0.634	1.000 (0.940, 1.060)	0.930	0.9309
ER- Breast cancer	4	3,092	119,039	FinnGen	1.085 (0.895, 1.315)	0.407	0.952 (0.806, 1.125)	0.562	0.948 (0.670, 1.341)	0.655	1.379 (0.643, 2.956)	0.211	0.267	1.080 (0.710, 1.650)	0.470	0.1584
Ovarian cancer	4	25,509	66,450	OCAC	1.038 (0.959, 1.124)	0.354	1.027 (0.935, 1.128)	0.582	0.992 (0.780, 1.263)	0.923	1.065 (0.731, 1.553)	0.546	0.773	1.040 (0.910, 1.190)	0.320	0.6299
Ovarian cancer	3	1,218	199,741	UKB	1.000 (0.999, 1.002)	0.762	1.000 (0.998, 1.002)	0.910	1.000 (0.996, 1.005)	0.933	1.000 (0.971, 1.030)	0.929	0.992	NA		
Ovarian cancer	4	719	123,579	FinnGen	0.818 (0.651, 1.028)	0.084	0.812 (0.606, 1.088)	0.163	0.808 (0.500, 1.306)	0.252	0.726 (0.245, 2.156)	0.334	0.650	0.820 (0.560, 1.190)	0.100	0.7646
High grade serous ovarian cancer	4	13,037	53,978	OCAC	1.039 (0.922, 1.171)	0.532	0.999 (0.886, 1.127)	0.988	0.992 (0.764, 1.288)	0.925	1.076 (0.542, 2.137)	0.692	0.828	1.040 (0.800, 1.350)	0.580	0.2653
Low grade serous ovarian cancer	4	1,012	41,953	OCAC	1.015 (0.748, 1.377)	0.925	1.040 (0.727, 1.486)	0.832	1.043 (0.423, 2.570)	0.891	0.721 (0.183, 2.844)	0.413	0.352	1.010 (0.520, 1.980)	0.930	0.3972
High grade and low grade serous ovarian cancer	4	14,049	54,990	OCAC	1.044 (0.941, 1.159)	0.417	1.000 (0.891, 1.121)	0.994	0.979 (0.765, 1.252)	0.802	1.052 (0.573, 1.930)	0.755	0.634	1.040 (0.830, 1.310)	0.480	0.3479
Clear cell ovarian cancer	4	1,366	42,307	OCAC	0.978 (0.722, 1.323)	0.883	0.977 (0.720, 1.325)	0.880	0.983 (0.468, 2.066)	0.946	1.153 (0.219, 6.067)	0.747	0.677	0.980 (0.500, 1.900)	0.890	0.262
Endometrioid ovarian cancer	4	2,810	43,751	OCAC	0.913 (0.714, 1.167)	0.468	0.881 (0.716, 1.084)	0.230	0.809 (0.463, 1.414)	0.314	0.736 (0.217, 2.497)	0.393	0.482	0.910 (0.530, 1.570)	0.520	0.1851
Invasive mucinous ovarian cancer	4	1,417	42,358	OCAC	1.028 (0.814, 1.299)	0.815	1.004 (0.754, 1.336)	0.979	0.964 (0.527, 1.763)	0.859	0.834 (0.272, 2.554)	0.557	0.460	1.030 (0.670, 1.580)	0.800	0.5917
Low grade and low malignant potential serous ovarian cancer	4	2,966	43,907	OCAC	0.928 (0.751, 1.146)	0.488	0.963 (0.777, 1.194)	0.734	1.088 (0.617, 1.920)	0.669	1.026 (0.313, 3.363)	0.935	0.722	0.930 (0.580, 1.480)	0.540	0.2855
Low malignant potential serous ovarian cancer	4	1,954	42,895	OCAC	0.880 (0.658, 1.176)	0.388	0.962 (0.735, 1.259)	0.778	1.051 (0.573, 1.928)	0.813	1.179 (0.302, 4.598)	0.655	0.407	0.880 (0.470, 1.660)	0.450	0.2031
Invasive and low malignant potential mucinous ovarian cancer	4	2,566	43,507	OCAC	1.013 (0.847, 1.212)	0.883	1.051 (0.857, 1.289)	0.632	1.076 (0.685, 1.689)	0.642	1.002 (0.427, 2.353)	0.993	0.954	1.010 (0.830, 1.240)	0.790	0.8455
Low malignant potential mucinous ovarian cancer	4	1,149	42,090	OCAC	1.011 (0.774, 1.320)	0.936	1.068 (0.779, 1.464)	0.681	1.083 (0.571, 2.053)	0.719	1.320 (0.373, 4.672)	0.445	0.414	1.010 (0.630, 1.620)	0.930	0.6433
Endometrial cancer	4	12,906	121,885	ECAC	1.047 (0.955, 1.148)	0.327	1.049 (0.944, 1.166)	0.376	1.057 (0.846, 1.319)	0.487	1.089 (0.694, 1.708)	0.501	0.716	1.050 (0.980, 1.120)	0.060	0.9621
Endometrial cancer	4	1,222	361,194	UKB	1.001 (1.000, 1.001)	0.160	1.001 (1.000, 1.001)	0.265	1.000 (0.999, 1.002)	0.560	1.001 (0.997, 1.004)	0.432	0.747	1.001 (1.000, 1.001)	0.018	0.9667
Endometrial cancer	4	1,053	123,579	FinnGen	1.210 (1.000, 1.464)	0.050	1.216 (0.954, 1.550)	0.114	1.231 (0.797, 1.903)	0.226	1.429 (0.577, 3.541)	0.232	0.467	1.210 (0.910, 1.620)	0.070	0.7655
Endometrial cancer (Endometrioid histology)	4	8,758	54,884	ECAC	1.055 (0.947, 1.176)	0.331	1.038 (0.918, 1.175)	0.548	1.036 (0.805, 1.333)	0.685	1.072 (0.629, 1.827)	0.631	0.900	1.060 (0.980, 1.130)	0.050	0.9653
Endometrial cancer (non-endometrioid histology)	4	1,230	36,677	ECAC	0.995 (0.766, 1.291)	0.967	0.993 (0.728, 1.353)	0.964	0.867 (0.436, 1.722)	0.555	0.782 (0.223, 2.736)	0.487	0.451	0.990 (0.660, 1.490)	0.960	0.6939
Cervical cancer	4	1,648	123,579	FinnGen	1.043 (0.895, 1.215)	0.592	0.995 (0.808, 1.224)	0.960	0.961 (0.656, 1.409)	0.764	1.044 (0.465, 2.344)	0.841	0.996	1.040 (0.770, 1.410)	0.600	0.4981
Cervical cancer	2	563	199,086	UKB	1.000 (0.999, 1.001)	0.973					NA					

NA - not available due to an insufficient number of instruments to perform the specified MR method, UKB=UK-Biobank, OCAC= Ovarian Cancer Association Consortium, BCAC= Breast Cancer Association Consortium, ECAC= Endometrial Cancer Association Consortium.

Table S7: Mendelian randomization analyses of phylloquinone on female-specific cancers using different methods with SNP–phylloquinone association estimates taken from models adjusting for triglycerides.

					**MR IVW**		**Weighted median**		**Weighted mode**		**MR-Egger**		
Outcome	SNPs	Cases	Total	Data sources	OR (95% CI)	P-val	OR (95% CI)	P-val	OR (95% CI)	P-val	OR (95% CI)	P-val	P_inter
Breast cancer	4	122,977	228,951	BCAC	0.989 (0.953, 1.027)	0.572	0.987 (0.943, 1.033)	0.572	0.973 (0.882, 1.074)	0.443	0.993 (0.842, 1.172)	0.875	0.918
Breast cancer	3	13,879	212,402	UKB	1.003 (0.999, 1.008)	0.134	1.004 (0.998, 1.009)	0.229	1.004 (0.990, 1.018)	0.360	1.005 (0.936, 1.080)	0.513	0.771
Breast cancer	4	8,401	123,579	FinnGen	0.956 (0.890, 1.027)	0.220	0.971 (0.891, 1.058)	0.501	0.974 (0.845, 1.122)	0.594	1.013 (0.751, 1.368)	0.866	0.432
ER+ Breast cancer	4	69,501	175,475	BCAC	0.990 (0.935, 1.049)	0.742	0.989 (0.934, 1.047)	0.702	0.980 (0.863, 1.112)	0.642	1.014 (0.751, 1.369)	0.864	0.739
ER+ Breast cancer	4	4,263	123,302	FinnGen	0.890 (0.810, 0.979)	0.017	0.885 (0.785, 0.997)	0.045	0.869 (0.706, 1.069)	0.120	0.860 (0.578, 1.281)	0.246	0.706
ER- Breast cancer	4	21,468	127,442	BCAC	0.996 (0.931, 1.067)	0.916	1.004 (0.925, 1.090)	0.922	1.009 (0.853, 1.194)	0.875	0.959 (0.708, 1.299)	0.610	0.594
ER- Breast cancer	4	3,092	119,039	FinnGen	1.103 (0.929, 1.311)	0.264	1.005 (0.845, 1.196)	0.951	0.942 (0.675, 1.315)	0.610	1.372 (0.861, 2.188)	0.100	0.142
Ovarian cancer	4	25,509	66,450	OCAC	1.041 (0.963, 1.126)	0.316	1.032 (0.939, 1.135)	0.511	1.005 (0.826, 1.224)	0.935	1.070 (0.758, 1.511)	0.486	0.728
Ovarian cancer	3	1,218	199,741	UKB	1.000 (0.999, 1.002)	0.787	1.000 (0.998, 1.002)	0.912	1.000 (0.996, 1.005)	0.939	1.000 (0.977, 1.024)	0.980	0.944
Ovarian cancer	4	719	123,579	FinnGen	0.828 (0.665, 1.032)	0.093	0.818 (0.625, 1.071)	0.144	0.826 (0.517, 1.321)	0.286	0.788 (0.313, 1.982)	0.382	0.809
High grade serous ovarian cancer	4	13,037	53,978	OCAC	1.038 (0.923, 1.169)	0.531	1.000 (0.884, 1.131)	0.997	0.993 (0.770, 1.281)	0.935	1.066 (0.569, 1.997)	0.704	0.855
Low grade serous ovarian cancer	4	1,012	41,953	OCAC	1.037 (0.767, 1.403)	0.813	1.060 (0.753, 1.490)	0.739	1.077 (0.500, 2.319)	0.779	0.830 (0.191, 3.615)	0.641	0.534
High grade and low grade serous ovarian cancer	4	14,049	54,990	OCAC	1.045 (0.944, 1.158)	0.396	1.004 (0.889, 1.133)	0.953	0.989 (0.773, 1.265)	0.894	1.053 (0.608, 1.824)	0.723	0.951
Clear cell ovarian cancer	4	1,366	42,307	OCAC	0.968 (0.719, 1.301)	0.828	0.956 (0.704, 1.298)	0.773	0.925 (0.449, 1.907)	0.755	1.075 (0.231, 5.007)	0.859	0.767
Endometrioid ovarian cancer	4	2,810	43,751	OCAC	0.930 (0.724, 1.196)	0.573	0.939 (0.742, 1.188)	0.600	0.971 (0.594, 1.585)	0.859	0.825 (0.228, 2.989)	0.587	0.690
Invasive mucinous ovarian cancer	4	1,417	42,358	OCAC	1.041 (0.826, 1.313)	0.733	1.031 (0.768, 1.384)	0.838	1.021 (0.579, 1.801)	0.914	0.907 (0.323, 2.542)	0.722	0.575
Serous ovarian cancer: low grade and low malignant potential	4	2,966	43,907	OCAC	0.947 (0.762, 1.177)	0.624	1.037 (0.827, 1.299)	0.754	1.077 (0.720, 1.612)	0.599	1.101 (0.383, 3.164)	0.734	0.554
Serous ovarian cancer: low malignant potential	4	1,954	42,895	OCAC	0.898 (0.667, 1.209)	0.479	1.007 (0.763, 1.330)	0.960	1.075 (0.640, 1.806)	0.688	1.225 (0.377, 3.976)	0.536	0.321
Mucinous ovarian cancer: invasive and low malignant potential	4	2,566	43,507	OCAC	1.022 (0.857, 1.220)	0.807	1.059 (0.859, 1.306)	0.589	1.070 (0.723, 1.583)	0.623	1.039 (0.476, 2.271)	0.851	0.926
Low malignant potential mucinous ovarian cancer	4	1,149	42,090	OCAC	1.010 (0.777, 1.313)	0.943	1.066 (0.769, 1.479)	0.702	1.079 (0.567, 2.055)	0.732	1.249 (0.397, 3.931)	0.491	0.453
Endometrial cancer	4	12,906	121,885	ECAC	1.049 (0.957, 1.149)	0.309	1.059 (0.950, 1.181)	0.303	1.064 (0.865, 1.309)	0.413	1.087 (0.715, 1.653)	0.481	0.713
Endometrial cancer	4	1,222	361,194	UKB	1.001 (1.000, 1.001)	0.168	1.000 (1.000, 1.001)	0.299	1.000 (0.999, 1.002)	0.509	1.001 (0.997, 1.004)	0.467	0.839
Endometrial cancer	4	1,053	123,579	FinnGen	1.193 (0.993, 1.433)	0.060	1.171 (0.931, 1.474)	0.178	1.177 (0.800, 1.731)	0.271	1.293 (0.599, 2.791)	0.287	0.650
Endometrial cancer (Endometrioid histology)	4	8,758	54,884	ECAC	1.052 (0.944, 1.172)	0.363	1.037 (0.916, 1.174)	0.568	1.035 (0.783, 1.366)	0.723	1.050 (0.639, 1.725)	0.713	0.991
Endometrial cancer (non-endometrioid histology)	4	1,230	36,677	ECAC	0.992 (0.767, 1.283)	0.951	0.998 (0.733, 1.358)	0.988	0.867 (0.448, 1.676)	0.540	0.808 (0.258, 2.526)	0.505	0.466
cervical cancer	4	1,648	123,579	FinnGen	1.027 (0.886, 1.190)	0.726	0.981 (0.820, 1.174)	0.837	0.968 (0.713, 1.314)	0.754	0.986 (0.492, 1.975)	0.937	0.795
cervical cancer	2	563	199,086	UKB	1.000 (0.999, 1.001)	0.986			NA				

NA - not available due to an insufficient number of instruments to perform the specified MR method, UKB=UK-Biobank, OCAC= Ovarian Cancer Association Consortium, BCAC= Breast Cancer Association Consortium, ECAC= Endometrial Cancer Association Consortium.

Table S8: Mendelian randomization analyses of phylloquinone on female-specific cancers using different methods and restricting the instrument to two clinically relevant SNPs (rs2192574 near to *CTNAA2* gene and rs2108622 near to *CYP4F2*).

Outcome	Data source	Method	SNP	OR (95% CI)	P-val
Breast cancer	BCAC	Inverse variance weighted	2	0.99 (0.94, 1.04)	0.68
Breast cancer	UKB	Wald ratio	1	1.00 (0.99, 1.01)	0.80
Breast cancer	FinnGen	Inverse variance weighted	2	0.90 (0.79, 1.04)	0.15
ER+ breast cancer	BCAC	Inverse variance weighted	2	0.98 (0.89, 1.08)	0.69
ER+ breast cancer	FinnGen	Inverse variance weighted	2	0.90 (0.75, 1.09)	0.28
ER- breast cancer	BCAC	Inverse variance weighted	2	1.01 (0.92, 1.12)	0.79
ER- breast cancer	FinnGen	Inverse variance weighted	2	0.93 (0.75, 1.15)	0.52
Ovarian cancer	OCAC	Inverse variance weighted	2	1.06 (0.93, 1.20)	0.37
Ovarian cancer	UKB	Wald ratio	1	1.00 (1.00, 1.00)	0.94
Ovarian cancer	FinnGen	Inverse variance weighted	2	0.81 (0.47, 1.39)	0.45
High grade serous ovarian cancer	OCAC	Inverse variance weighted	2	1.09 (0.82, 1.43)	0.56
Low grade serous ovarian cancer	OCAC	Inverse variance weighted	2	1.20 (0.79, 1.82)	0.38
High grade and low grade serous ovarian cancer	OCAC	Inverse variance weighted	2	1.10 (0.89, 1.37)	0.39
Clear cell ovarian cancer	OCAC	Inverse variance weighted	2	1.02 (0.49, 2.11)	0.96
Endometrioid ovarian cancer	OCAC	Inverse variance weighted	2	0.98 (0.62, 1.56)	0.93
Invasive mucinous ovarian cancer	OCAC	Inverse variance weighted	2	1.12 (0.79, 1.58)	0.52
Low grade and low malignant potential serous ovarian cancer	OCAC	Inverse variance weighted	2	0.97 (0.72, 1.32)	0.85
Low malignant potential serous ovarian cancer	OCAC	Inverse variance weighted	2	0.87 (0.47, 1.63)	0.66
Invasive and low malignant potential mucinous ovarian cancer	OCAC	Inverse variance weighted	2	1.05 (0.81, 1.37)	0.69
Low malignant potential mucinous ovarian cancer	OCAC	Inverse variance weighted	2	0.96 (0.57, 1.60)	0.86
Endometrial cancer	ECAC	Inverse variance weighted	2	1.04 (0.91, 1.18)	0.56
Endometrial cancer	UKB	Inverse variance weighted	2	0.96 (0.72, 1.27)	0.76
Endometrial cancer	FinnGen	Inverse variance weighted	2	1.05 (0.74, 1.49)	0.79
Endometrial cancer (endometrioid histology)	ECAC	Inverse variance weighted	2	1.04 (0.89, 1.21)	0.64
Endometrial cancer (non-endometrioid histology)	ECAC	Inverse variance weighted	2	1.18 (0.81, 1.71)	0.40
Cervical cancer	FinnGen	Wald ratio	1	1.00 (1.00, 1.00)	0.92
Cervical cancer	UKB	Inverse variance weighted	2	1.00 (1.00, 1.00)	0.56

UKB=UK-Biobank, OCAC= Ovarian Cancer Association Consortium, BCAC= Breast Cancer Association Consortium, ECAC= Endometrial Cancer Association Consortium.

Table S9: Statistical power and minimum detectable odds ratio at 80% power for the association between phylloquinone and four female-specific cancers.

Cancer types	Cases (total)	Statistical power in percentage			Minimum detectable OR
		Log odds ratio=0.10/OR=1.11	Log odds ratio=0.50/OR=1.65	Log odds ratio=0.70/OR=2.00	
Breast					
BCAC	122,977 (228,951)	99.99	100.00	100.00	1.06
UKB	13,879 (212,402)	69.47	100.00	100.00	1.13
FinnGen	8,401 (123,579)	48.34	100.00	100.00	1.16
Meta-analysis	145, 257 (549,075)	100.00	100.00	100.00	1.04
Ovarian					
OCAC	25,509 (66,450)	77.57	100.00	100.00	1.12
UKB	1,218 (199,741)	11.40	96.50	99.95	1.46
FinnGen	719 (123,579)	8.37	82.59	98.20	1.63
Meta-analysis	27,446 (389,770)	93.36	100.00	100.00	1.08
Endometrial					
ECAC	12,906 (121,885)	64.39	100.00	100.00	1.13
UKB	1,222 (361,194)	11.44	96.58	99.95	1.45
FinnGen	1,053 (123,579)	10.39	93.85	99.84	1.49
Meta-analysis	15,181 (606,658)	75.10	100.00	100.00	1.12
Cervical					
UKB	563 (199,086)	7.40	72.85	94.90	1.73
FinnGen	1,648 (123,579)	13.88	99.20	100.00	1.39
Meta-analysis	2,211 (322,665)	17.26	99.91	100.00	1.32
Coagulation factor IX*	(10,708)	66.00	100.00	100.00	1.13

*= positive control outcome, OR=Odds ratio, UKB=UK-Biobank, OCAC= Ovarian Cancer Association Consortium, BCAC= Breast Cancer Association Consortium, ECAC= Endometrial Cancer Association Consortium.

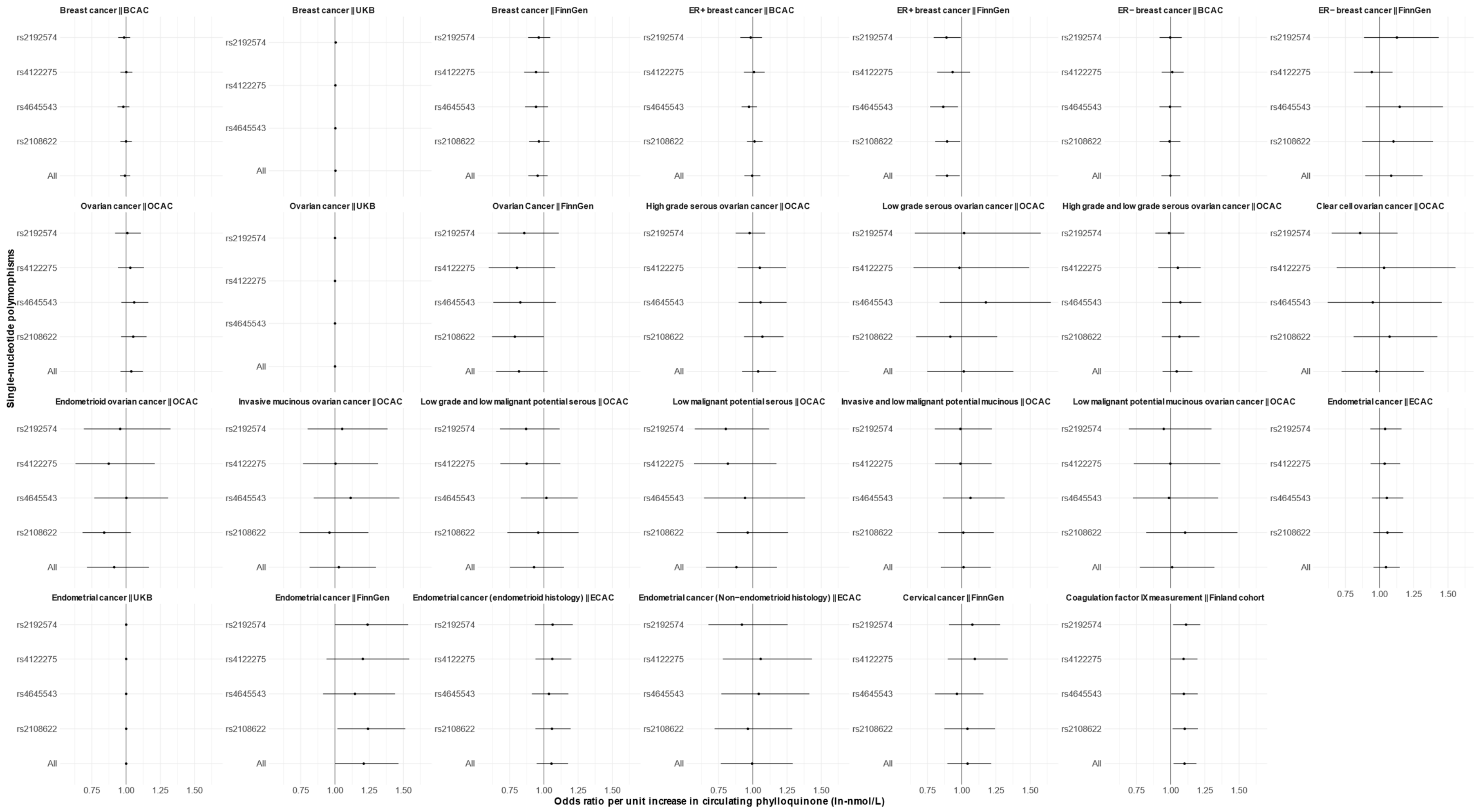

Figure S1: Leave-one-out sensitivity analysis for the association between circulating phylloquinone, four female-specific cancers, and the coagulation factor IX measurement as the positive control.
